# Mitochondrial DNA-triggered innate immune response: mechanisms and diseases

**DOI:** 10.1038/s41423-023-01086-x

**Published:** 2023-11-07

**Authors:** Ming-Ming Hu, Hong-Bing Shu

**Affiliations:** 1 Department of Infectious Diseases, Medical Research Institute, Zhongnan Hospital of Wuhan University, Frontier Science Center for Immunology and Metabolism, Taikang Center for Life and Medical Sciences, College of Life Sciences, Wuhan University, Research Unit of Innate Immune and Inflammatory Diseases, Chinese Academy of Medical Sciences, Wuhan, 430072 China; 2 Research Unit of Innate Immune and Inflammatory Diseases, Chinese Academy of Medical Sciences, Wuhan, 430072 China

**Keywords:** Mitostress, Virus, Innate immunity, Disease, MITA/STING, Immunology, Mechanisms of disease

## Abstract

Various cellular stress conditions trigger mitochondrial DNA (mtDNA) release from mitochondria into the cytosol. The released mtDNA is sensed by the cGAS-MITA/STING pathway, resulting in the induced expression of type I interferon and other effector genes. These processes contribute to the innate immune response to viral infection and other stress factors. The deregulation of these processes causes autoimmune diseases, inflammatory metabolic disorders and cancer. Therefore, the cGAS-MITA/STING pathway is a potential target for intervention in infectious, inflammatory and autoimmune diseases as well as cancer. In this review, we focus on the mechanisms underlying the mtDNA-triggered activation of the cGAS-MITA/STING pathway, the effects of the pathway under various physiological and pathological conditions, and advances in the development of drugs that target cGAS and MITA/STING.

## Cytoplasmic nucleic acid-sensing pathways

The innate immune arm of the immune system forms the first line of host defense against pathogen infection, and it is initiated via the recognition of conserved microbial structures by cellular pattern recognition receptors (PRRs) [1–6]. After viral infection, invading viral nucleic acids, such as viral RNA (vRNA) or DNA (vDNA), are recognized by PRRs, which initiate signaling pathways that ultimately induce the expression of type I interferon (IFN), proinflammatory cytokine, and other antiviral effector genes [3–7]. These downstream effectors inhibit viral replication, induce apoptosis in infected cells, and promote activation of the adaptive immune response, leading to antiviral immune responses [1–6]. In contrast, viruses evolve multiple strategies to evade the host immune response to maintain their survival and persistent infection [7–14]. The relative power of these two opposing forces determine the eventual outcomes of the viral infection of a host.

After RNA virus infection of mammalian cells, vRNA invading the cytoplasm is sensed by RIG-I-like receptors (RLRs), which include RIG-I and MDA5 [1–3, 7]. After sensing vRNA, RLRs undergo conformational changes, oligomerization, and then translocation to mitochondria [15, 16], where they interact with Virus-Induced Signaling Adaptor (VISA, also called MAVS, Cardif, and IPS-1) [17–20]. VISA then recruits WDR5, TRAF and cIAP proteins, the kinases TBK1 and IKK, and the transcription factors IRF3 and NF-κB [21–26]. In these complexes, activated TBK1 and IKK phosphorylate and activate IRF3 and NF-κB, respectively, leading to their translocation into the nucleus and expression of downstream antiviral genes [1, 2]. The RLR-VISA axis activity is regulated by cofactors, distinct posttranslational modifications and regulators of posttranscriptional modifications to ensure efficient initiation of innate antiviral immunity and its timely termination in the late phase of infection [16, 23, 24, 27–43].

After DNA virus infection of mammalian cells, vDNA is sensed by a widely expressed enzyme called cyclic GMP–AMP synthase (cGAS) [4, 44–46]. Various studies have demonstrated that cGAS not only recognizes a wide range of microbial DNA but also senses mitochondrial DNA (mtDNA) and cellular nuclear DNA (nDNA) aberrantly localized to the cytosol after infection or under stress or pathological conditions [47–54]. After binding to cytosolic DNA, cGAS undergoes phase separation and forms “DNA-cGAS” liquid droplets to achieve optimal activation, after which it catalyzes the synthesis of cyclic GMP–AMP (cGAMP) from the substrates GTP and ATP [46, 55]. Newly synthesized cGAMP binds to Mediator of IRF3 Activation (MITA) (also known as STING and ERIS) in the endoplasmic reticulum (ER), which promotes MITA/STING oligomerization and trafficking from the ER to perinuclear punctate structures [56–62]. During the cellular trafficking processes, MITA/STING recruits TBK1 and IRF3, leading to IRF3 phosphorylation, dimerization and translocation into the nucleus to drive the transcription of type I IFN genes [56, 60, 63–66]. Additionally, NF-κB is activated by the MITA/STING-associated complex, leading to the transcription of inflammatory cytokine genes [67]. The cGAS-MITA/STING axis is extensively regulated by cofactors. For example, ZCCHC3, G3BP1 and PCBP1 promote cGAS binding to DNA [68–70]; ZDHHC1 and sulfated glycosaminoglycans (sGAGs) are important for cGAMP-triggered dimerization/oligomerization of MITA/STING [59, 61]; and iRhom2, SNX8, VPS34 and ARMH3 are critically involved in MITA/STING trafficking and activation [60, 62, 71]. The cGAS-MITA/STING axis is also extensively regulated by posttranslational modifications, such as cGAS acetylation [72, 73] and phosphorylation [74–77], MITA/STING phosphorylation [38, 78–80], and polyubiquitination [81–90]. These positive and negative regulating modification of cGAS-MITA/STING axis components ensures efficient and proper innate immune responses to DNA viruses. Because of the critical importance of the cGAS-MITA/STING pathway in the innate immune response to DNA viruses, it is extensively targeted by DNA viruses to enable viral immune evasion [13, 91–103]. Interestingly, in addition to induction of the innate immune response, activation of the cGAS-STING axis has also been reported to cause other effects, such as controlling autophagy [104], mRNA translation [105], IRF3-independent function [106], which contribute to several pathological processes, including clearance of invading pathogens, cell senescence and organ fibrosis, as well as the antitumor response.

**Fig. 1:**
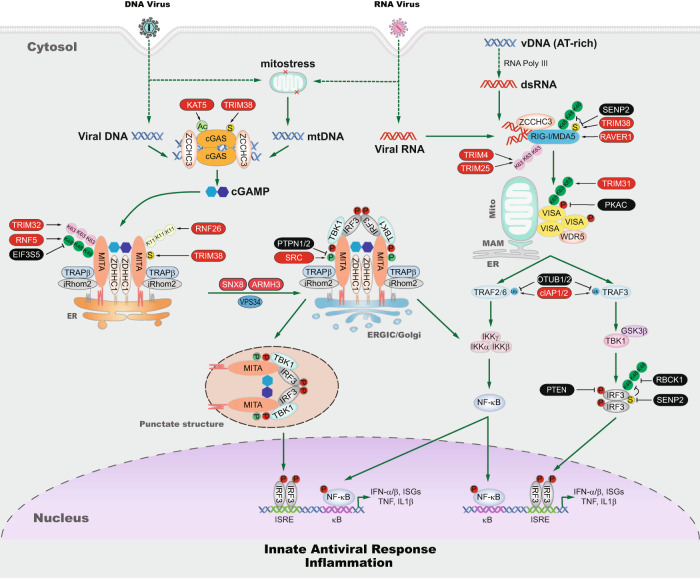
Cytoplasmic nucleic acid-sensing pathways. After RNA virus infection of mammalian cells, the invading vRNA is sensed by RLRs, which include RIG-I and MDA5, in the cytoplasm. After sensing vRNA, RLRs are translocated to mitochondria, where they interact with VISA. VISA then recruits WDR5 and TRAF proteins, the kinases TBK1 and IKK, and the transcription factors IRF3 and NF-κB. In these complexes, activated TBK1 and IKK phosphorylate and activate IRF3 and NF-κB, respectively, leading to their translocation into the nucleus and expression of downstream antiviral genes. After DNA virus infection of mammalian cells, vDNA is sensed by cGAS. After binding to cytosolic DNA, cGAS catalyzes the synthesis of cGAMP from the substrates GTP and ATP. cGAMP binds to MITA/STING at the endoplasmic reticulum (ER), which promotes MITA/STING oligomerization and trafficking from the ER to perinuclear punctate structures. During trafficking, MITA/STING recruits TBK1 and IRF3, leading to IRF3 phosphorylation, dimerization and translocation into the nucleus to drive the transcription of type I IFN genes. Additionally, NF-κB is activated by the MITA/STING-associated complex, leading to the transcription of inflammatory cytokine genes. The cGAS-MITA/STING axis is extensively regulated by cofactors, such as ZCCHC3, G3BP1, PCBP1, ZDHHC1, iRhom2 and ARMH3. Some AT-rich viral DNA sequences can be transcribed to vRNA by RNA polymerase III, and they are sensed by RIG-I to initiate innate immune signaling. Infection with certain RNA or DNA viruses causes mtDNA release from mitochondria into the cytosol, which triggers the innate immune response via the cGAS-MITA/STING axis. Cytoplasmic RNA- and DNA-sensing pathways are common used in host cells and antagonize either RNA or DNA viruses. RIG-I retinoic acid-inducible gene I; MDA5 melanoma differentiation-associated protein 5; VISA virus-induced signaling adaptor; WDR5 WD repeat-containing protein 5; TRAF TNF receptor-associated factor; TBK1 TANK-binding kinase 1; IKK inhibitor of nuclear factor kappa-B kinase; IRF3 interferon regulatory factor 3; cGAS cyclic GMP-AMP synthase; MITA mediator of IRF3 activation; STING stimulator of interferon gene; ZCCHC3 zinc finger CCHC domain-containing protein 3; G3BP1 GTPase activating protein (SH3 domain) binding protein 1; PCBP1 Poly(rC)-binding protein 1; ZDHHC1 zinc finger DHHC domain-containing protein 1; iRhom2 inactive rhomboid protein 2; ARMH3 Armadillo-like helical domain-containing protein 3

It has been established that infection with RNA viruses is mainly sensed by RIG-I and MDA5, while infection with DNA viruses is mostly sensed by cGAS. It has been shown that *Rig-i*^*-/-*^ mouse embryo fibroblasts (MEFs) do not produce type I IFNs in response to Sendai virus (SeV), vesicular stomatitis virus (VSV), influenza virus, paramyxovirus, Japanese encephalitis virus (JEV), and hepatitis C virus (HCV) [107–109], while *Mda5*^-/-^ MEFs do not respond to picornaviruses such as encephalomyocarditis virus (EMCV) and Theiler’s virus [110]. Moreover, *Rig-i*^-/-^ and *Mda5*^-/-^ mice show increased susceptibility to infection with VSV and EMCV, respectively [111]. These studies suggest that different RNA viruses can be sensed by distinct RLR family members. Various studies indicated that *cGas*^-/-^ and *Mita*^-/-^ knockout mice produce much lower levels of type I IFNs and other cytokines after DNA virus infection and are highly susceptible to infection with DNA viruses, such as herpes simplex virus-1 (HSV-1) and vaccinia virus (VACV), suggesting that the cGAS-MITA/STING pathway is critically important for the innate immune response to DNA viruses [44, 68]. However, various studies have shown that deficiency in cGAS or MITA/STING did not completely abolish the expression of antiviral effector genes triggered by DNA viruses [44]. It has been shown that some AT-rich DNA viruses can be transcribed into vRNA by RNA polymerase III, which is sensed by RIG-I, initiating innate immune signaling [112–115]. On the other hand, various reports have demonstrated that the expression of type I IFNs and downstream interferon-stimulating genes (ISGs) following infection with certain RNA viruses is abrogated in *cGas*^-/-^ and *Mita*^-/-^ knockout cells, suggesting that the cGAS-MITA/STING pathway also participates in the innate immune response to certain RNA viruses [48, 116–120]. Clearly, the cytoplasmic RNA- and DNA-sensing pathways are common in host cells and antagonize RNA or DNA viruses (Fig. 1). Moreover, infection with certain RNA viruses causes mtDNA release from mitochondria into the cytosol, which triggers the innate immune response via the cGAS-MITA/STING axis [48, 121]. Various studies have demonstrated that mtDNA is released after cells undergo a specific type of stress, and this mtDNA is critically involved in inflammatory and autoimmune responses [122, 123]. These studies suggest that mtDNA release under various stress conditions may be a convergent and common mechanism underlying cellular defense.

## mtDNA is released from mitochondria into the cytosol under stress conditions

Mitochondria are important organelles in eukaryotic cells, as they synthesize ATP and metabolites [124]. Mitochondria can respond to external or endogenous stresses that trigger mitochondrial autophagy (mitophagy), mtDNA release, and apoptosis, and thus, they ultimately regulate of the survival or death of stressed cells [124–126]. Although the mechanisms underlying mitophagy and apoptosis have been extensively studied in recent decades, the mechanisms and effects of mtDNA release have only been studied in recent years. In cells under stress, mtDNA is released into the cytoplasm where it is sensed as a danger signal and thus activates a variety of signaling pathways in cells, including the cGAS-mediated innate immune response [48], absent in melanoma 2 (AIM2)- or NLR family pyrin domain-containing 3 (NLRP3)-mediated inflammation [127–131] and genomic DNA damage repair [132]. Recently, Z-DNA-binding protein 1 (ZBP1) has been reported to stabilize Z-form mtDNA and act as a cooperative partner for the cGAS response to mitochondrial genome instability [133]. In this article, we focus on the mechanisms of mtDNA release and its effects on the innate immune response under stress conditions or after viral infection.

**Fig. 2:**
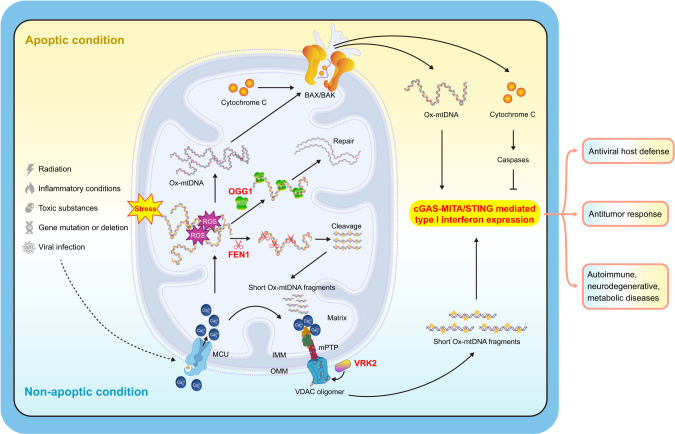
Mechanisms of mtDNA release and its effects on innate immune response. The release of mtDNA from mitochondria into the cytosol is triggered by different factors, including radiation exposure, microbial infection, inflammatory conditions, toxic substances or drugs, and gene mutation or deletion. Under apoptotic conditions, mitochondrial contents, including oxidized mtDNA and cytochrome c, are released from the mitochondrial matrix into the cytosol, leading to the activation of the cGAS-MITA/STING pathway and caspases, respectively. Activated caspases cleave cGAS and IRF3 to suppress type I IFN expression. Under nonapoptotic conditions, only oxidized mtDNA is released to activate the cGAS-MITA/STING pathway. In this context, mtDNA undergoes fragmentation into 500–650 bp fragments by the DNA enzyme FEN1 before release into the cytosol, while OGG1 can mediate mtDNA glycosylation and thereby protect mtDNA from fragmentation. Under some circumstances (e.g., viral infection), MCU-mediated Ca^2+^ import into mitochondria is important for the initiation of mtDNA oxidation and MPTP opening. mtDNA-induced activation of the cGAS-MITA/STING pathway and subsequent type I IFN expression play important roles in antiviral host defense, antitumor response, and development of autoimmune, neurodegenerative and metabolic diseases. cGAS cyclic GMP-AMP synthase, MITA mediator of IRF3 activation, STING stimulator of interferon gene, FEN1 Flap endonuclease 1, OGG1 8-oxoguanine DNA glycosylase, VRK2 vaccinia related kinase 2, MCU mitochondrial calcium uniporter, MPTP mitochondrial permeability transition pore, OMM outer mitochondrial membrane, IMM inner mitochondrial membrane

Circular mtDNA in vertebrates is maternally inherited and encodes eleven subunits of the mitochondrial electron transport chain and two subunits of ATP synthase. These proteins provide the wiring for the oxidation phosphorylation (OXPHOS) system [134, 135]. mtDNA also encodes 22 tRNAs and two rRNAs that are essential for mRNA translation in the mitochondrial matrix. The other mitochondrial proteins, including the various factors needed for mtDNA replication, repair and gene expression, are encoded by cellular DNA and are targeted to or imported into mitochondria [124]. In addition to acting as coding genes, mtDNA can be released into the cytosol under various cellular stress conditions (Fig. 2), leading to innate immune and inflammatory responses [48]. The release of mtDNA from mitochondria to the cytosol can be triggered by different factors, including radiation exposure, microbial infection, inflammatory conditions, toxic substances or drugs, and gene mutation or deletion [122, 123]. For example, radiation therapy can cause mitochondrial stress (mitostress) in tumor cells, which results in the release of mitochondrial contents, including cytochrome c and mtDNA, into the cytosol [136–138]. Viral infection can cause mitochondrial stress and release of mtDNA via Ca^2+^ uptake by the mitochondrial calcium uniporter (MCU) in a variety of cells [48, 118, 121]. The proinflammatory cytokines TNF and IL-1β have been reported to trigger mtDNA release in a variety of cells, such as myeloid cells, fibroblasts and epithelial cells, leading to activation of cGAS [139, 140]. A variety of foreign substances, including liposomes and crystalline silicon, have been reported to induce mtDNA release and thus activate innate immune and inflammatory responses [74, 141]. In addition, the release of mtDNA from mitochondria to the cytosol can be triggered by mutation or deletion of certain mitochondrion-related genes, which are involved in maintaining mitochondrial structure, mtDNA stabilization or mitophagy [48, 142–144]. These studies indicate that mtDNA release from mitochondria to the cytosol is triggered by divergent factors in different cell types, suggesting that mtDNA release is a basic and common cellular response to stress conditions.

## Molecular mechanisms of mtDNA release

mtDNA is normally wrapped with Transcriptional Factor A Mitochondrial (TFAM) in the mitochondrial matrix [124]. Studies have shown that TFAM-coated mtDNA is stable and more resistant to ROS oxidation, while newly synthesized naked mtDNA is more susceptible to oxidation and leakage into the cytosol [128, 145]. Studies have shown that mtDNA undergoes oxidation and then fragmentation into 500–650 bp fragments by the DNA enzyme Flap Endonuclease 1 (FEN1) before its release into the cytosol, while 8-oxoguanine DNA glycosylase (OGG1) can mediate mtDNA glycosylation and thereby protect mtDNA from fragmentation [145] (Fig. 2). Studies have proven that oxidized mtDNA induces a greater immunostimulatory effect both in vitro and in vivo [146, 147]. However, the molecular mechanisms of the oxidation and fragmentation of mtDNA under different stress conditions need to be further elucidated.

Mitochondria are bilayer membranous organelles that consist of the outer mitochondrial membrane (OMM) and inner mitochondrial membrane (IMM) [124]. mtDNA needs to cross the IMM and OMM for export from the mitochondrial matrix into the cytosol. Recent studies indicate that major IMM pores include the mitochondrial permeability transition pore (MPTP), which is a nonspecific mitochondrial channel that is activated by Ca^2+^ influx [49, 145, 148], and inner mitochondrial membrane (IMM) herniation, which is triggered by oligomerization of Bcl-2-associated X protein (BAX) and Bcl-2 homologous antagonist/killer (BAK) [50]. Under oxidative stress, voltage-dependent anion channel 1 (VDAC1) located at the OMM can oligomerize and mediate mtDNA release. In the presence of mtDNA fragments, VDAC1 oligomerization is accelerated [149]. VDAC1 was initially considered a component of the MPTP; however, an analysis of VDAC1-deficient mice showed that VDAC1 deficiency exerted no marked effects on MPTP formation, which indicated that VDAC1 is dispensable for MPTP formation or a compensatory system consisting of other VDAC family members is involved [150]. How MPTP opening and VDAC1 oligomerization synergistically mediate mtDNA release is still unknown. Of course, VDAC2/3 might compensate for VDAC1 deficiency. During apoptosis, the formation of BAX/BAK macropores causes OMM permeabilization and mtDNA extrusion [50]. Under certain stress conditions, mtDNA may cross the IMM and OMM via one or more of these pores and eventually leak into the cytosol. The detailed mechanisms explaining how these pores are formed and regulated need further investigation. Recently, vaccinia-related kinase 2 (VRK2) was identified as a regulator of VDAC1 oligomerization [121]. Virus-induced mtDNA release into the cytosol is markedly impaired in VRK2-deficient cells [121]. It has also been suggested that rapid Ca^2+^ uptake via the mitochondrial calcium uniporter (MCU) precedes MPTP opening and subsequent VDAC1 oligomerization [121, 145]. Additionally, Prohibitin 1 (PHB1) has been reported to regulate mtDNA release by inhibiting mPTP opening [151]. Whether other events, such as mitochondrial ROS accumulation, trigger MPTP opening and how stress conditions signal these events are unanswered questions. Furthermore, even though BAX/BAK oligomerization can lead to mtDNA release during apoptosis, the release of mtDNA mediated by VDAC but not BAX/BAK oligomerization has been shown to be involved in most diseases (see below).

## Physiological and pathological effects of mtDNA release

mtDNA released into the cytosol is sensed by the widely expressed DNA sensor cGAS in a DNA sequence-independent manner. The cGAS response results in the synthesis of cGAMP and subsequent MITA/STING-dependent signaling events, leading to the expression of type I IFNs and various inflammatory cytokines, including TNF superfamily members, interleukins and chemokines [45, 63]. Notably, most of the physiological and pathological effects of mtDNA release are mediated by these cytokines (Fig. 2).

### mtDNA release contributes to host defense against viral infection

Various studies have demonstrated that infection with either DNA or RNA viruses triggers mtDNA release from mitochondria into the cytosol, where it activates the cGAS-MITA/STING axis, leading to the activation of downstream antiviral effectors [48, 116, 121, 152, 153]. Depletion of mtDNA by ethidium bromide (EB) or dideoxycytidine (ddC) downregulates the transcription of *IFNB1* and other antiviral genes after HSV-1 infection [48, 121]. Deficiency of VRK2, which is a regulator of VDAC1 oligomerization and mtDNA release, renders mice more susceptible to infection with both the DNA virus HSV-1 and the RNA virus EMCV [121]. Although rapid induction of type I IFNs limits virus propagation, a sustained increase in the levels of type I IFNs in the late phase of infection is associated with aberrant inflammation and poor clinical outcomes. It has been suggested that in patients with COVID-19, the cGAS-MITA/STING pathway is critical to the type I IFN immunopathology of extrapulmonary complications in lung endothelial cells after mtDNA release [11, 154, 155]. There is accumulating evidence showing that mtDNA sensing by the cGAS-MITA/STING pathway plays a critical role in antiviral host defense, but how viral infection triggers Ca^2+^ import into mitochondria via MCU to induce mitostress and how MPTP-VDAC oligomer-mediated mtDNA release is regulated after viral infection are not fully understood. Furthermore, whether specific viral mechanisms suppress mtDNA release to enable immune evasion is also an unanswered question.

### Radiation-induced mtDNA release benefits antitumor therapy

Previous studies have demonstrated that ionizing radiation-mediated tumor regression is dependent on the activation of the cGAS-MITA/STING axis in dendritic cells (DCs) [138]. Accordingly, activation of MITA/STING by cGAMP enhances radiation-triggered antitumor immunity [138]. Recently, studies have also demonstrated that mtDNA released into the cytosol of irradiated tumor cells failed to activate the cGAS-MITA/STING pathway due to caspase 3/9-mediated suppression of this signaling pathway [136, 156]. In this context, caspases have been reported to cleave cGAS and IRF3 to suppress type I IFN activation [157]. The combination of radiation, a caspase inhibitor and an anti-PD-L1 antibody promoted antitumor therapy [136]. Additionally, it has been reported that mtDNA drives abscopal responses to radiation exposure that are inhibited by autophagy [137]. Autophagy-deficient cells secrete increased amounts of type I IFNs, suggesting that autophagy inhibitors may serve as potential drugs for increasing the efficacy of radiation therapy in cancer patients [137].

### mtDNA release causes autoimmune, neurodegenerative and metabolic diseases

Activation of IRF3 and induction of type I IFNs protect the host against infection and cancer, but excessive IFN responses triggered by self-DNA (including mtDNA) under sterile conditions cause autoinflammatory conditions such as Aicardi–Goutières syndrome (AGS), STING-associated vasculopathy of infancy (SAVI) and systemic lupus erythematosus (SLE) [158, 159]. Neutrophil extracellular traps (NETs) have been implicated in autoimmunity, and NETs enriched with oxidized mtDNA are interferongenic and contribute to lupus-like disease [160]. Another study also indicated that mtDNA fragments released following VDAC oligomerization promoted lupus-like disease [149]. The VDAC inhibitor VBIT-4 reduced mtDNA release, the IFN response and disease severity in a mouse model of SLE [149].

Recently, studies have demonstrated that deregulation of the cGAS-MITA/STING axis is involved in multiple sterile inflammatory diseases, such as myocardial infarction, heart failure, cardiac hypertrophy, aortic aneurysm and dissection, obesity, and nonalcoholic fatty liver diseases [161–169]. This is because of the large loads of damage-associated molecular patterns, including mtDNA and/or DNA in extracellular vesicles liberated during recurrent injury to metabolic cellular organelles and tissues, which are sensed by the cGAS-MITA/STING pathway [170, 171]. Furthermore, although the cGAS-MITA/STING pathway-mediated immune response is often neuroprotective, excessive or sustained activation of this pathway in the brain causes neuroinflammation and neurodegeneration [172, 173]. Therefore, targeting the cGAS-MITA/STING pathway can have potential therapeutic benefits in patients with one of several neurodegenerative disorders, including Alzheimer’s disease [174], Parkinson’s disease [175], and amyotrophic lateral sclerosis (ALS) [49].

## Intervention of diseases by targeting the cGAS-MITA/STING pathway

Since deregulation of the mtDNA-cGAS-MITA/STING pathway causes aberrant innate immune and inflammatory responses and pathological effects, great efforts have been made to identify strategies for selective modulation of the cGAS-MITA/STING axis in various diseases, and these strategies include identification of agonists of the cGAS-MITA/STING axis to use as vaccine adjuvants or as anticancer and antiviral immunostimulatory agents, as well as identification of selective inhibitors of the axis to use as potential drugs for treating inflammatory and autoimmune diseases. Specific agents targeting cGAS and MITA/STING are summarized in Table 1.

**Table 1:** Small-molecule compounds that target MITA/STING and cGAS

	Biological effects	Refs
MITA/STING agonists		
ADU-S100, BMS-986301, MK-1454	For treatment of advanced solid tumors with monotherapy and combined ICIs	[181, 201, 202]
Di-ABZI	Caused regression of solid tumors	[182, 203]
C11, BNBC	Activate MITA/STING-mediated immune responses and block replication of multiple alphavirus types	[183, 184]
MSA-2	Exhibits antitumor immune activity and synergizes with anti-PD-1 antibody treatments	[185]
SR-717	Exhibits antitumor activity	[186]
Kitacinnamycin 8	Increases cGAMP-induced IFN-β expression	[204]
α-Mangostin, G10	Activate the MITA/STING-TBK1-IRF3 pathway and repolarize M2 macrophages into M1 macrophages	[187, 188]
DSDP	Induces MITA/STING-dependent cytokine production and inhibits the replication of a variety of viruses	[189]
MITA/STING inhibitors		
C-176, C-178, H-151	Exerts therapeutic effects in *Trex1*^−/−^ tumor model mice	[193]
NO2-Fas	Exhibits suppressive activity in THP-1 cells and BMDMs	[194]
Tetrahydroisoquinolines	Inhibit cGAMP- induced IFN-β secretion in THP-1 cells	[204]
Astin C	Exhibits suppressive activity in mouse and human fibroblasts	[191]
cGAS inhibitors		
RU.521	Suppresses cGAS activity in macrophages	[196]
PF-06928215	Suppresses cGAS activity in THP-1 cells	[195]
Hydroxychloroquine, quinacrine	Inhibit IFN-β production in cells and block cGAS-DNA binding	[198, 199]
Suramin	Inhibits IFN-I production in THP-1 cells	[200, 205]

### MITA/STING agonists

Activating the cGAS-MITA/STING pathway can enhance the antitumor immune response [176]. In addition, MITA/STING agonists can be used as adjuvants to develop vaccines against certain infectious diseases [177]. To date, most MITA/STING activators have been synthetic cyclic dinucleotides (CDNs), such as ADU-S100 [178], BMS-986301 (Clinical Trials.gov ID: NCT03956680) and MK-1454 [179]. Intratumoral injection of these CDNs induces intense antitumor T-cell immune responses and generates immune memory, leading to complete tumor regression as well as prevention of distal metastasis of lung cancers [180, 181].

Considering the high polarity and proteolytic tendency of the abovementioned cyclic dinucleotide agonists, their clinical application potential is limited. In recent years, nonnucleotide derivatives have gained prominence due to their high specificity and effectiveness. For example, amide compounds, such as amide benzimidazole (ABZI) and its derivatives, Compounds 16 *g*, 24b, and 24e, N-(methylcarbamoyl)-2-[5] phenylacetamide (C11), 6-bromo-n-(naphthalen-1-yl)-benzo (d), and dioxole-5-carboxamide (BNBC), have been identified as human MITA/STING agonists [182–184]. These compounds, when given intravenously, exerted potent antitumor effects in mice bearing subcutaneous tumors. Furthermore, other compounds, such as MSA-2 [185] and SR-717 [186], have also been identified as MITA/STING nonnucleotide agonists. These compounds can induce IFN-β production in tumors and a long-lasting antitumor immune response and can also exert synergistic effect when administered with an anti-PD-1 antibody therapy [185, 186]. MSA-2 and SR-717 show the potential for clinical application because of their oral availability characteristics and simplified administration mode. Additionally, flavonoid compounds have been reported to be MITA/STING agonists; they include α-mangostin, G10, and dispiro diketopiperazine (DSDP) [187–189]. The effects of these agonists on antitumor activities need to be investigated.

### MITA/STING inhibitors

Two main approaches have been utilized to identify MITA/STING inhibitors. The first approach involves the design of molecules that target the CDN-binding site, thereby functioning as competitive inhibitors of MITA/STING activation. This class of MITA/STING inhibitors mainly includes tetrahydroisoquinolines [190] and astin C [191]. The second approach is to identify molecules that bind to either the Cys88 or the Cys91 residue of the human MITA/STING protein, each of which is a target for palmitoylation [192]. Tetrahydroisoquinolines bind to MITA/STING dimers in 2:2 ratio and thus inhibit cGAMP-induced IFN-β secretion from THP-1 cells [190]. Astin C is a natural product that binds competitively to the CDN site and blocks the recruitment of IRF3 to the MITA/STING signalosome. Furthermore, astin C inhibits the expression of type I IFNs in *Trex1*^-/-^ BMDMs and mice [191]. To date, several different chemicals targeting MITA/STING palmitoylation residues have been reported, including nitrofurans (C-176 and C-178) [193], indole urea (H-151) [193] and nitro fatty acids [194]. Mice administered C-176 exhibited markedly reduced production of serum type I IFNs induced by MITA/STING agonists. Additionally, pretreatment of *Trex1*^-/-^ mice with C-176 led to a significant reduction in type I IFN levels and the number of inflammatory signatures in the heart [193]. Intraperitoneal administration of H-151 inhibited the systemic cytokine response triggered by a MITA/STING agonist [193]. Additionally, certain nitro fatty acids reduced type I IFNs in response to DNA viral infection in both THP-1 cells and BMDMs [194].

### cGAS inhibitors

Two classes of cGAS inhibitors have been developed for the treatment of inflammatory and autoimmune diseases. The first class of cGAS antagonists bind to the enzymatic active site, resulting in competition with the ATP or GTP substrate or the product cGAMP. The second class of cGAS antagonists block DNA binding to cGAS, thereby inhibiting the initial step in cGAS activation. The catalytic site inhibitors of cGAS include PF-06928125 [195], RU.521 [196] and G150 [197], and the action of most has been validated only in vitro. For example, RU.521 has been shown to be a selective inhibitor of cGAS that reduced the *Ifnb1* mRNA level in bone marrow-derived macrophages (BMDMs) from *Trex1*^-/-^ mice [196]. Inhibitors that disrupt the DNA-binding activity of cGAS are mainly antimalarial drugs. It has been reported that antimalarial drugs, including hydroxychloroquine and quinacrine, can be used for SLE treatment since these drugs suppress IFN-β expression by blocking the cGAS–dsDNA interaction [198, 199]. It has also been reported that suramin can bind to cGAS and disrupt the formation of the cGAS–dsDNA complex [200]. In vivo studies on the functions of these selective cGAS inhibitors are needed to validate their roles in the intervention of inflammatory and autoimmune diseases in the future.

## Concluding remarks

Studies in recent years have established critical roles for mtDNA release in cellular defense against various stress conditions, including stress caused by infection with various types of viruses, deregulation of autophagy and introduction of DNA aberrations. Studies have identified components involved in mtDNA release from mitochondria into the cytosol after viral infection or under other stress conditions. However, more studies are needed to identify the common and distinct mechanisms of mtDNA release under different stress conditions. Released mtDNA is sensed by cGAS, which triggers MITA/STING-mediated innate immune responses. However, deregulation of the mtDNA-cGAS-MITA/STING axis activity leads to inflammatory and autoimmune diseases as well as cancer. In recent years, small molecules targeting MITA/STING or cGAS have been developed for potential application in cancer immunotherapy or the treatment of inflammatory and autoimmune diseases. Further studies on the molecular mechanisms underlying the mtDNA-cGAS-MITA/STING axis will certainly provide a more comprehensive understanding of cellular defense against viral infection and other stress conditions and help in the development of novel strategies for the intervention of serious human diseases, including inflammatory/autoimmune diseases and cancer.
